# Immunotherapy in Biliary Tract Cancers: Current Standard-of-Care and Emerging Strategies

**DOI:** 10.3390/cancers15133312

**Published:** 2023-06-23

**Authors:** Justin H. Lo, Rajiv Agarwal, Laura W. Goff, Thatcher R. Heumann

**Affiliations:** Division of Hematology and Oncology, Department of Medicine, Vanderbilt University Medical Center, Nashville, TN 37232, USA

**Keywords:** biliary tract cancer, cholangiocarcinoma, gallbladder adenocarcinoma, immunotherapy, immune checkpoint inhibition, combination therapy, perioperative immunotherapy, immune microenvironment

## Abstract

**Simple Summary:**

Biliary tract cancers (BTCs), comprising cancers of the bile ducts (cholangiocarcinoma) as well as gallbladder cancers, continue to be challenging to treat. Cancer immunotherapy strategies harness the immune system to help to attack cancer cells. A number of immunotherapy approaches have been or are currently being studied in BTCs, including immune checkpoint inhibitors (ICIs) that block immune-suppressing signals, immune-stimulating agonists, modified T cells that target tumor components (CAR-T), and cancer vaccines. Here, we discuss the use of immunotherapy on its own or in combination with (1) chemotherapy, (2) drugs that target specific tumor proteins, or (3) blood-vessel-targeted treatments. At present, the combination of an ICI with chemotherapy is established as the standard of care, first-line treatment for BTCs that have spread to distant parts of the body or that cannot be removed surgically. Ongoing work will help to explore further approaches and scenarios in which immunotherapy may positively impact the management of BTCs.

**Abstract:**

Biliary tract cancers (BTCs), comprising intrahepatic, perihilar, and distal cholangiocarcinoma as well as gallbladder adenocarcinoma, continue to be challenging to manage. Conventional chemotherapy regimens for advanced disease are limited in both options and benefits, and more effective perioperative regimens are also needed. Over the last decade, immunotherapy has had a profound impact on the management of many solid tumor types, particularly in using immune checkpoint inhibition to enable a tumor-directed T cell response. Immunotherapy administered on its own has had limited utility in BTCs, in part due to a hostile immune microenvironment and the relative infrequency of biomarker-based tumor-agnostic indications for immunotherapy. However, immunotherapy in conjunction with chemotherapy, molecularly targeted therapies, and/or anti-angiogenic therapies has gained traction, supported by evidence that these agents can impart favorable immunomodulatory effects on the tumor microenvironment. The TOPAZ-1 trial led to the first BTC-specific immunotherapy approval, establishing the combination of durvalumab with gemcitabine and cisplatin as the preferred first-line treatment for advanced or metastatic disease. Recently, the KEYNOTE-966 trial showed positive results for the combination of pembrolizumab with gemcitabine and cisplatin in the same setting, adding further evidence for the addition of immune checkpoint inhibition to the standard chemotherapy backbone. Meanwhile, advances in the molecular profiling of BTCs has contributed to the recent proliferation of molecularly targeted therapeutics for the subset of BTCs harboring alterations in *IDH1*, *FGFR2*, MAP kinase signaling, *HER2*, and beyond, and there has been great interest in investigating combinations of these agents with immunotherapy. Emerging immunotherapy strategies beyond immune checkpoint inhibition are also being studied in BTCs, and these include immunostimulatory receptor agonists, Wnt signaling modulators, adoptive cell therapy, and cancer vaccines. A large number of trials are underway to explore promising new combinations and immune-targeted strategies, offering opportunities to expand the role of immunotherapy in BTC management in the near future.

## 1. Introduction

Biliary tract cancers (BTCs) consist of intrahepatic cholangiocarcinoma (iCCA), extrahepatic cholangiocarcinoma (eCCA, further subdivided into perihilar and distal cholangiocarcinoma), and gallbladder adenocarcinoma (GBA). There are approximately 18,000 new cases of BTCs annually in the United States, where the incidence of iCCA, in particular, has been increasing in recent years [1,2,3,4]. BTCs continue to be very challenging to manage, with the American Cancer Society estimating a 5-year survival of 9% for iCCA and 11% for eCCA at all stages combined based on SEER data [5]. Traditionally, systemic therapy options for the adjuvant treatment or management of unresectable and metastatic disease have not distinguished between the different subtypes of BTCs, owing in part to their relative rarity. However, the molecular and phenotypic heterogeneity of BTCs has been increasingly appreciated in the nascent age of precision medicine, as, for instance, in the major advances in targeted therapies for alterations in iCCA. Moreover, a more nuanced understanding of the tumor microenvironment has led to efforts to capitalize upon the modulation of the tumor microenvironment of BTCs as the basis for formulating new combination therapy approaches.

Immunotherapy has proliferated in the last decade as an important systemic treatment modality, with immune checkpoint inhibitors (ICIs), in particular, now playing a major role in the management of a variety of solid tumors. With tumor-agnostic biomarker-based indications for ICIs being rare in BTCs, and early studies using ICIs alone showing limited promise, the major breakthrough thus far has featured the combination of an ICI with standard-of-care first-line chemotherapy. This approach was pioneered through the TOPAZ-1 trial that confirmed the benefit of adding durvalumab to gemcitabine plus cisplatin in the first-line treatment of advanced BTCs [6], leading to the first BTC-specific FDA approval of immunotherapy in 2022. While the second-line treatment of advanced BTCs has been revolutionized by molecularly targeted therapeutics in recent years, the options available for cases without actionable alterations have very limited efficacy, prompting considerable investigation into a possible role of immunotherapy in this setting. Finally, there are limited options and evidence for the use of perioperative treatment to improve outcomes of the curative-intent resection of BTCs, representing another space where immunotherapy may have an impact in the future. In this review, we will discuss the current and upcoming landscape of immunotherapy in the management of BTCs, including the use of ICIs alone and in combination with chemotherapy, small-molecule therapeutics, and anti-angiogenic agents, as well as immunotherapeutics beyond ICIs.

## 2. Background

### 2.1. The Immune Microenvironment of Biliary Tract Cancers

The tumor microenvironment (TME) plays an important role in the response to immunotherapies. The BTC microenvironment broadly includes immune cells, endothelial and lymphatic cells, and cancer-associated fibroblasts (CAFs), as well as the acellular components, including the extracellular matrix (ECM) [7]. Together, stromal components compose the majority of tissues in many BTCs, with one study finding that the stroma comprises ~73% of pancreaticobiliary tumors, by far the highest of any tested tumor type [8].

The array of immune cells in the TME of BTCs is diverse, including tumor-infiltrating lymphocytes (B lymphocytes, cytotoxic CD8^+^ T lymphocytes, CD4^+^ regulatory T lymphocytes (Tregs), and CD4^+^ effector T lymphocytes), NK cells, macrophages (both tumor-associated macrophages and, for iCCA, Kupffer cells), and tumor-associated neutrophils (TANs) [7]. There is considerable heterogeneity among the immune profiles of BTCs, though in a study that classified 368 iCCA tumors, by far the most common immune phenotype (45%) was that of an “immune desert”, in which the expression of innate and adaptive immune signatures was uniformly depressed [9]. Unfortunately, as discussed below, multiple tumor-intrinsic and tumor-extrinsic factors in BTCs can contribute to an immunosuppressed environment that leads to primary resistance to immunotherapy.

Biliary tract tumors can influence the immune components in the surrounding microenvironment in a number of direct and indirect ways. Oncogenes relevant to BTCs such as *IDH1*, *FGFR2*, *KRAS*, *BRAF*, and *HER2* are known to lead to modulation of the immune microenvironment. The distribution of these oncogenic alterations in BTC subtypes and the corresponding targeted pharmacologic interventions are summarized in Table 1.

**Table 1:** Common molecular alterations in BTCs. Oncogenic alterations are presented according to BTC subtype, along with corresponding targeted agents and immunotherapy combinations that have been or are currently being studied in clinical trials (see also Table 2 and Table 3). Rates of alterations adapted from Valle et al. [10].

Oncogene	iCCA	eCCA	GBA	Targeted Agents	Combinations with IO in BTC Trials
*IDH1*/*IDH2*	4.9–36%	0–7.4%	1.5%	Ivosidenib *	Ivosidenib + nivolumab
*FGFR1, 2,* or *3* fusion/mut./amp.	11–45%	-	3%	Pemigatinib ** Futibatinib **	Pemigatinib + pembrolizumab
MAP kinase pathway: *KRAS* mut. *BRAF* mut.	8.6–24.2% 3–7.1%	8.3–42% 3%	4–13% 1–5.9%	- Dabrafenib	*Mutation agnostic:* Cobimetinib + atezolizumab Cobimetinib + atezolizumab + varlilumab
*ERBB2* or *3* (HER2 or 3) amp.	7%	11–17%	9.8–19%	Trastuzumab Pertuzumab	Trastuzumab + nivolumab + chemo

*: Full FDA approval for BTCs; **: accelerated FDA approval for BTCs. Abbreviations: amp.: amplification; mut.: mutations.

For example, *IDH1*-mutant tumors can lead to the increased production and release of D-2-hydroxyglutarate and have an inhibitory effect on other cells, including CD4^+^ and CD8^+^ T lymphocytes [11,12]. Consequently, in mouse models, the combination of IDH1 small-molecule inhibitors and anti-PD-1 [12] or anti-CTLA-4 [13] immune checkpoint inhibitors resulted in improved outcomes compared to IDH1 inhibition alone. 

Aberrant FGFR signaling at the tumor level can lead to the downregulation of antigen-presenting MHC II molecules and, in some tumor types, upregulate PD-L1 [14]. Additionally, the FGFR response to FGF in immune cells produces an overall immunosuppressive effect by upregulating PD-1 on effector T cells, promoting the survival of regulatory T cells via STAT5 phosphorylation, and polarizing macrophages towards the M2 phenotype [14]. In genetically engineered mouse models of *FGFR2*- and *p53*-mutant lung cancer, treatment with the pan-FGFR inhibitor erdafitinib resulted in decreased amounts of Tregs and increased CD4^+^ and CD8^+^ effector T cell presence. Furthermore, erdafitinib treatment in combination with an ICI produced a significant improvement in OS in these preclinical models compared to either agent alone [15].

Oncogenic *KRAS* upregulates the expression of TLR4, leading to downstream IL-1β production, which, in turn, promotes M2 macrophage polarization and the accumulation of Th17 cells and myeloid-derived suppressor cells (MDSCs), ultimately suppressing CD8^+^ cytotoxic T cell activation [16]. *KRAS* inhibition in immunocompetent mouse tumor models reversed this phenotype and resulted in an increased M1/M2 macrophage polarization ratio as well as increased tumor-infiltrating CD4^+^ and CD8^+^ T cells [17].

Additionally, in the case of *HER2*, the targeting of overexpressed or amplified HER2 receptors with trastuzumab produces an anti-tumor effect, in part through eliciting an increased adaptive and innate immune response [18], and there is preclinical evidence for synergy between anti-HER2 and anti-PD-1 therapy [19], as well as clinical evidence from other cancer types such as gastric adenocarcinoma [20]. The clinical evidence for combining molecularly targeted strategies with ICIs in BTCs is discussed in Section 3.3.

**Figure 1:**
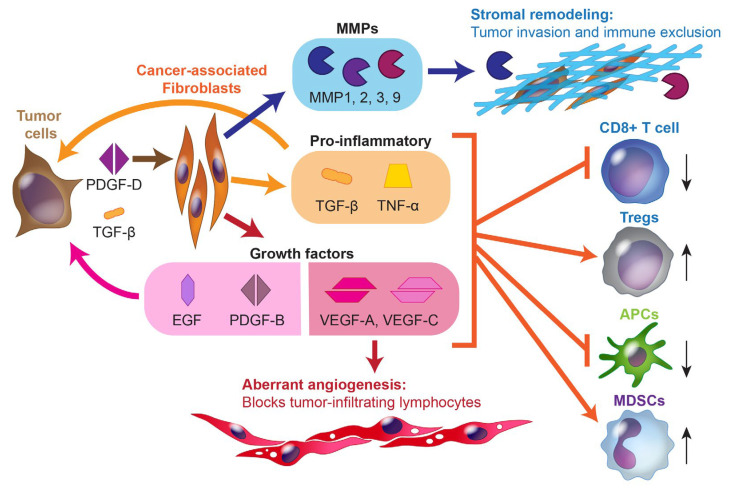
Selected signaling pathways between biliary tract tumor cells, cancer-associated fibroblasts, vasculature, and tumor immune cells. Abbreviations: APCs: antigen-presenting cells; MDSCs: myeloid-derived suppressor cells; MMPs: matrix metalloproteinases.

Beyond the effects of molecular alterations within biliary tract tumor cells themselves, these malignant cells also secrete PDGF-D that helps to recruit large numbers of CAFs to the tumor environment through PDGFR-β signaling [21], while the malignant production of FGF, PDGF, and TGF-β maintains the CAFs in a persistently activated state [22], ultimately producing the desmoplastic stromal reaction characteristic of BTCs. The CAFs in BTCs are thought to originate from hepatic stellate cells (for iCCAs), portal fibroblasts, and/or circulating mesenchymal cells [3]. When activated, these CAFs release (1) pro-inflammatory compounds including TGF-β, IL-1β, and prostaglandin E2 that reinforce production by the tumor cells themselves; (2) ECM-remodeling factors including MMP1, MMP2, MMP3, and MMP9; and (3) growth factors including EGF, PDGF-B, and angiogenic factors, such as VEGF-A and VEGF-C [7]. These VEGF isoforms, in turn, engage multiple VEGF receptors (VEGFR1-3) and neuropilin-1/2, which are located at various points on endothelial and lymphatic cells, immune cells, and tumor cells themselves [23]. These signaling mechanisms and their consequences are depicted in Figure 1.

VEGF-mediated signaling has many consequences on the immune environment. Most directly, it can lead to the activation of FOXP3^+^ Tregs and MDSCs while inhibiting cytotoxic T lymphocytes and the maturation of antigen-presenting dendritic cells; this has an overall immunosuppressive/tolerogenic effect [23,24]. VEGF is also well-known as the major driver of tumor angiogenesis and lymphatic recruitment. While the tumor-associated vasculature does provide some degree of access to tumor cells for systemic therapies, these blood vessels are poorly formed, with gaps, large fenestrations, and a lack of uniformity or standard vascular hierarchy. This presents a physical barrier to T lymphocyte penetration of the tumor, with one study finding an inverse correlation between CD31+ vessel density and both CD4^+^ and CD8^+^ T lymphocytes in cholangiocarcinoma [25].

Systemic therapies can produce profound changes in the TME. Antiangiogenic therapies targeting VEGF or VEGF receptors can have the effect of normalizing tumor blood vessels, removing a barrier to infiltration by immune cells, and reversing the immunosuppressive effects of VEGF signaling on dendritic cells and T lymphocytes. As detailed in the discussion of oncogene-related immune modulation above, molecularly targeted therapies can undo immunosuppression that is downstream of oncogenic tumor signaling pathways. This effect is likely to extend beyond the immediate impact of these targeted therapies on the tumor cell themselves, since the targeted proteins are often active in elements of the TME as well. For example, FGFR family inhibition will not only have an effect of FGFR2 fusion on cholangiocarcinoma cells but also interrupt FGF/FGFR signaling interactions that lead to CAF growth, Treg activation, M2 TAM polarization, and angiogenesis [14]. Finally, cytotoxic chemotherapy can also lead to immunomodulation. In the case of cisplatin, a key component in the current first-line therapy for BTCs, multiple mechanisms of immunomodulation have been proposed, including (1) the upregulation of MHC class I expression, (2) recruitment and proliferation of cytotoxic T lymphocytes and other immune effector cells, (3) reduction in the levels of MDSCs and Tregs, and (4) promotion of the lytic activity of cytotoxic effectors [26]. These mechanisms underlie the recent interest in combination therapies that leverage these responses to contrasting systemic therapies in order to enhance the response of BTCs to immunotherapies, as discussed in Section 3.2, Section 3.3 and Section 3.4 below.

Another important consideration for immune-targeted therapeutics is the potential for acquired tumor resistance in the subset of BTCs that initially respond to immunotherapy. Acquired resistance has been best-characterized in tumors treated with ICIs, and the mechanisms include tumor-intrinsic and tumor-extrinsic changes. Tumor-intrinsic mechanisms include the depletion of neoantigens, downregulation of tumor neoantigen presentation, acquisition of oncogenic mutations that confer immunosuppressive properties in the TME, and phenotypic changes to tumor cells such as transdifferentiation or epithelial–mesenchymal transition [27,28]. The major tumor-extrinsic mechanism is compensation for PD-L1/PD-1 and/or CTLA-4/CD80/86 blockade through alternate immune checkpoints such as LAG-3, TIM-3, TIGIT, and VISTA [28]. Thus, strategies that can help to overcome tumor-intrinsic acquired resistance to ICI-based monotherapy or dual therapies include combinations with chemotherapy, oncogene-targeted therapies, antiangiogenic therapies, immunostimulatory antibodies (e.g., CD27 or CD40), adoptive T or NK cell transfer, and cancer vaccination, as discussed in Section 3.2, Section 3.3, Section 3.4 and Section 3.5.

### 2.2. Landscape of Chemotherapy in Advanced Biliary Tract Cancers

For over a decade prior to the incorporation of immunotherapy into BTC management, the standard-of-care first-line regimen was gemcitabine plus cisplatin based on the seminal phase III ABC-02 trial published in 2010 [29]. Patients were randomized to gemcitabine/cisplatin or gemcitabine monotherapy, with the doublet regimen achieving a median progression-free survival (mPFS) of 8.0 months and median overall survival (mOS) of 11.7 months, a statistically significant improvement compared to 5.0 months and 8.1 months for gemcitabine alone. For the doublet arm, the objective response rate (ORR) was 26.1% and the disease control rate (DCR) was 81.4%.

Despite promising results in a single-arm phase II trial [30], the augmentation of gemcitabine/cisplatin with nab-paclitaxel did not result in a statistically significant improvement in mOS compared to the doublet alone, based on recently reported data from the phase III SWOG 1815 trial [31]. Likewise, the phase II PRODIGE 38 AMEBICA trial randomized patients to modified FOLFIRINOX (5-fluorouracil/leucovorin (5-FU/LV) plus irinotecan plus oxaliplatin) or standard-of-care gemcitabine/cisplatin. There was no statistical difference between outcomes for these two regimens, with an mPFS and mOS of 6.2 and 11.7 months for mFOLFIRINOX and 7.4 and 13.8 months for gemcitabine/cisplatin, respectively [32]. Thus, gemcitabine plus cisplatin continues to be the chemotherapy backbone of choice for BTCs, and the key studies combining immunotherapy with chemotherapy in the first-line setting have used this regimen as the chemotherapy component, as detailed in Section 3.2.

Second-line therapy in advanced BTCs is a rapidly evolving space. The widening array of targeted therapies available for BTCs with actionable alterations are discussed in Section 3.3. For patients without actionable alterations, treatment options include FOLFOX (5-FU/LV plus oxaliplatin) and 5-FU/LV plus nanoliposomal (nal-)irinotecan. In the phase III ABC-06 trial, FOLFOX resulted in an ORR of 4.9% with a significant but clinically marginal median overall survival (mOS) benefit of 6.2 months compared to 5.3 months with supportive care alone [33]. The mPFS for FOLFOX was 4.0 months. The NIFTY phase IIb trial more recently compared 5-FU/LV with or without nal-irinotecan in the second-line setting. The blinded-independent-central-review (BICR)-assessed mPFS was 7.1 months with nal-irinotecan vs. 1.4 months without nal-irinotecan [34]; the differences were smaller in the updated extended follow-up data, in which the masked ICR-assessed mPFS was reported as 4.2 and 1.7 months, respectively [35]. The investigator-assessed mOS was 8.6 months with nal-irinotecan compared to 5.3 months without nal-irinotecan. Given the modest outcomes of standard-of-care second-line chemotherapy, there has been interest in the role of immunotherapy in the second line, particularly as standalone therapeutics (Section 3.1) or in combination with second-line chemotherapy (Section 3.2), antiangiogenic therapy (Section 3.4), or targeted small-molecule therapeutics (Section 3.3). 

## 3. Immunotherapy in Advanced Biliary Tract Cancers

### 3.1. Immune Checkpoint Inhibitors as Monotherapy or Doublet Therapy

**Figure 2:**
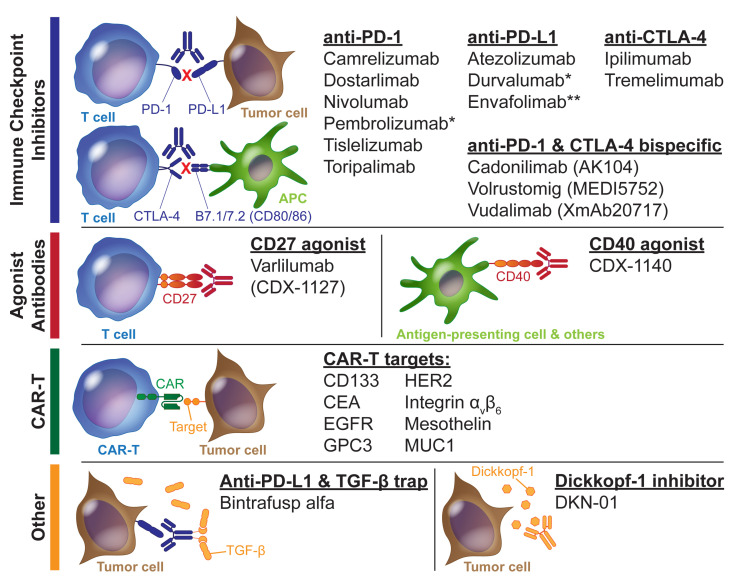
Immunotherapy agents established or being studied in BTCs, categorized according to mechanism of action. Agents are listed alphabetically. *: FDA-approved. Durvalumab approved in combination with gemcitabine/cisplatin. Pembrolizumab, currently approved for TMB-H disease. **: Subcutaneous administration (all others are intravenous).

Immune checkpoint inhibitors (ICIs) function by blocking one or more surface protein interactions that regulate the adaptive immune response, chiefly the interaction between PD-L1 (tumor and stromal cells) and PD1 (T cells) that prevents tumor cell killing by cytotoxic T lymphocytes or between CTLA-4 (T cells) and CD80/CD86 (antigen-presenting cells) that leads to T cell anergy. ICIs have become a mainstay in the modern management of many solid tumors, with approved indications on their own and in combination with chemotherapy or antiangiogenic agents. A reference list of ICIs and other immunotherapies being studied in BTCs is provided in Figure 2, alongside their mechanism of action. Table 2 lists results from selected completed trials using immunotherapy in BTCs.

To date, ICIs, on their own, have had a limited role in the clinical management of BTCs. A small minority of BTCs qualify for immunotherapy based on tumor-type-agnostic biomarkers, with only around 2% being microsatellite-instability-high (MSI-H) and approximately 4% being tumor-mutation-burden-high (TMB-H, in this study defined as ≥17 mutations/megabase) [36].

Several studies have investigated ICI therapy in the second-line setting for advanced BTCs. The phase Ib KEYNOTE-028 and phase II KEYNOTE-158 trials treated patients with pembrolizumab monotherapy in the second line, including 24 and 104 patients with BTCs, respectively [37]. The objective response rates (ORRs) were 13.0% for KEYNOTE-028 and 5.8% for KEYNOTE-158. PD-L1 positivity, defined as a combined positive score (CPS) ≥ 1%, was required for KEYNOTE-028, while KEYNOTE-158 enrolled PD-L1-positive (CPS ≥ 1%), -negative (CPS < 1%), and unknown cases. The assays used to quantify CPS differed between the two studies. In KEYNOTE-158, PD-L1 status did not have a significant effect on PFS, and partial responses were seen in both PD-L1-positive and -negative patients. A phase II study of nivolumab monotherapy in the second line also showed modest efficacy, with an ORR of 10.9% according to a central independent review. The mPFS was 3.7 months, while the mOS was 14.2 months. Notably, in this study, PD-L1 positivity (defined as a tumor-cell-only positive score ≥1%) was associated with an improved PFS.

The combination of nivolumab and ipilimumab (weight-based dosing) was investigated in the nonrandomized CA209-538 phase II trial (NCT02923934) [38]. The analysis of the BTC subgroup showed an ORR of 23% and disease control rate (DCR) of 44%, while the median PFS was 2.9 months and mOS was 5.7 months. The phase I NCT01938612 trial tested two regimens in the dose expansion phase—durvalumab plus tremelimumab (weight-based dosing rather than the fixed dosing used in more contemporary trials) and durvalumab monotherapy—in a heavily pretreated advanced BTC population [39]. Grade 3 or higher treatment-related adverse events were similar between the two groups at 23% and 19%, respectively, and a partial response (PR) was seen in 7/65 (10.8%) patients receiving the doublet and 2/42 (4.8%) patients receiving durvalumab alone. The IMMUNO-BIL/PRODIGE 57 randomized phase II trial (NCT03704480) was originally intended to study the use of second-line durvalumab plus tremelimumab with or without paclitaxel in platinum-resistant BTCs [40]. However, the chemoimmunotherapy arm was closed early due to dose-limiting toxicity. The interim results for the doublet immunotherapy arm showed an ORR of 9.7% (including two complete responses) and a DCR of 40.8%; the median PFS was 2.5 months and median OS was 8.0 months. It is noteworthy that this regimen initially utilized tremelimumab dosed at 75 mg for four cycles, whereas the protocol was later amended to a 300 mg single dose to match the STRIDE regimen dosing used in hepatocellular carcinoma [41]. Durvalumab and tremelimumab have since been investigated further in other chemotherapy combinations, as discussed in Section 3.2. Dual checkpoint inhibition in the second line has thus shown some efficacy, though to date, there have not been any direct comparisons to chemotherapy regimens, which remain the standard of care for fit patients without actionable alterations.

Moving beyond traditional ICI monoclonal antibodies, the bispecific anti-PD1/anti-CTLA-4 monoclonal antibody XmAb20717 (vudalimab) is being studied in a single-arm phase II trial (NCT05297903) for second-line treatment; the primary endpoint is ORR, and secondary outcomes will include PFS, OS, and ORR stratified according to whether or not the patient received prior immunotherapy (Table 3 lists selected clinical trials in progress). Additional bispecific ICIs, including cadonilimab and MEDI5752 (volrustomig), are being studied in combination with antiangiogenics and are discussed in Section 3.4. 

Even though ICIs have been incorporated into standard first-line management as part of chemoimmunotherapy regimens, combination and novel approaches to immune checkpoint inhibition merit further study in the second-line setting, including investigations answering important questions regarding sequencing after first-line therapies that include single-ICI therapeutics. This is discussed further in the subsequent section.

### 3.2. ICIs in Combination with Chemotherapy

The greatest success thus far in utilizing immunotherapy for BTCs has come in the form of adding immune checkpoint blockade to standard first-line chemotherapy. Preclinical research has shown the potential for chemotherapy, particularly cisplatin [26] and the gemcitabine/cisplatin combination [42], to cause immunomodulation in the tumor environment, providing a rationale for combining an ICI with the ABC-02 regimen. Indeed, while cisplatin is conventionally thought of as immunosuppressive owing to its effects on total blood counts, at the tumor level, it has been shown to have effects such as recruiting effector cells, upregulating MHC class I expression to promote tumor antigen presentation, and increasing cytotoxic T cell lytic function [26].

The phase II randomized BilT-01 trial studied (A) gemcitabine/cisplatin with nivolumab and (B) ipilimumab with nivolumab in the first line for advanced/metastatic BTCs, comparing the 6-month PFS to a historical control (the ABC-02 trial) [43]. The chemoimmunotherapy arm showed a 6-month PFS of 59.4%, while the doublet immunotherapy arm showed only 21.2%, with neither arm significantly exceeding the historical control threshold of 59%. The authors concluded that the chemoimmunotherapy arm had a similar performance to gemcitabine/cisplatin alone, while the immunotherapy-only arm appeared inferior. However, it is possible that the selection of 6-month PFS as the primary endpoint may have been problematic, given the timeline for immunotherapy benefit seen in the trials discussed below, and the authors noted a 2-year OS of 35.4% in the gemcitabine/cisplatin plus nivolumab arm compared to the historical control of ~15–22% with chemotherapy alone, hinting at the possibility of delayed benefit in a subset of patients.

**Table 2:** Selected clinical trial results involving immunotherapy in BTCs.

NCT Number	Trial Name	Ph.	Treatment	Ln.	Prim. Endpoint	Met Endpt.	ORR	PFS (mo.)	OS (mo.)	Ref.
Immune-checkpoint-inhibitor-only monotherapy and combination therapy										
NCT02054806	KEYNOTE-028	I	Pembrolizumab	2nd	ORR	-	13.0%	1.8	5.7	[37]
NCT02628067	KEYNOTE-158	II	Pembrolizumab	2nd	ORR	-	5.8%	2.0	7.4	[37]
NCT02829918	-	II	Nivolumab	2nd	ORR	-	10.9%	3.7	14.2	[44]
NCT02923934	CA209-538	II	Ipilimumab/nivolumab	2nd	DCR		23.1%	2.9	5.7	[38]
NCT01938612	-	I	Durvalumab Durvalumab/tremelimumab	2nd	DLT/AE	-	4.8% 10.8%	1.5 1.6	8.1 10.1	[45]
NCT03704480	IMMUNO-BIL/ PRODIGE 57	II	Durvalumab/tremelimumab (interim)	2nd	OS (6 mo)	-	9.7%	2.5	8.0	[40]
Immune checkpoint inhibitors plus chemotherapy										
NCT03875235	TOPAZ-1	III	Gem/cis + durvalumab Gem/cis	1st	OS	Yes	26.7% 18.7%	7.2 5.7	12.8 11.5	[6]
NCT04003636	KEYNOTE-966	III	Gem/cis + pembrolizumab Gem/cis	1st	OS	Yes	28.7% 28.5%	6.5 5.6	12.7 10.9	[46]
NCT03101566	BilT-01	II	Gem/cis + nivolumab Ipilimumab/nivolumab	1st	PFS	No	22.9% 3.0%	6.6 3.9	10.6 8.2	[43]
NCT03046862	-	II	Gem/cis + durvalumab + tremelimumab Gem/cis + durvalumab Gem/cis, then gem/cis + durvalumab + tremelimumab	1st	ORR	Yes	70.2% 72.3% 50.0%	12.3 11.8 12.8	18.7 20.2 15.0	[47]
Immune checkpoint inhibitors plus molecularly targeted therapy										
NCT02393248	FIGHT-101	I/II	Pemigatinib + pembrolizumab	2nd	AEs, ORR	-	No BTC-only analysis			[48]
NCT03201458	-	II	Cobimetinib + atezolizumab Atezolizumab	2nd	PFS	Yes	3.3% 2.8%	3.7 1.9	-	[49]
NCT03639935	BilT-02	II	Rucaparib + nivolumab maintenance after platinum-based chemotherapy	Mnt.	PFS4	No	6.4%	4.6 *	15.9 *	[50,51]
Immune checkpoint inhibitors plus antiangiogenics										
NCT03895970	-	II	Lenvatinib + pembrolizumab	2nd	ORR, DCR, PFS	-	25.0%	4.9	11.0	[52]
NCT03797326	LEAP-005	II	Lenvatinib + pembrolizumab	2nd	ORR	-	9.7%	6.1	8.6	[53]
NCT03475953	REGOMUNE	II	Regorafenib + avelumab	2nd	ORR	-	13.8%	2.5	11.9	[54]
NCT02443324	JVDF	I	Ramucirumab + pembrolizumab	2nd	DLT	-	3.8%	1.6	6.4	[55]
NCT04677504	IMBrave-151	II	Gem/cis + atezolizumab + bevacizumab Gem/cis + atezolizumab	1st	PFS		24.1% 25.3%	8.4 7.9	-	[56]
NCT03951597	-	II	Gem/ox + lenvatinib + toripalimab	1st	ORR	Yes	80.0%	10.2	22.5	[57,58]
Other										
NCT02699515	-	I	Bintrafusp alfa	2nd	ORR	-	20.0%	2.5	12.7	[59]
NCT03833661	INTR@PID BTC 047	II	Bintrafusp alfa	2nd	ORR	No	10.7%	1.8	7.6	[60]
NCT02375880	-	I	Gem/cis + DKN-01	1st	ORR	-	21.3%	8.7	12.4	[61]
NCT01869166	-	I	EGFR CAR-T	2nd	AEs	-	5.9%	-	-	[62]
NCT03358849	-	I/II	Allogeneic NK cells + pembrolizumab	2nd	DLT	-	17.4%	-	-	[63]
-	-	I	WT1 peptide vaccine + gemcitabine		Safety	-	-	-	9.5	[64]
-	-	I	MUC1 peptide vaccine		Safety	-	0.0%	-	-	[65]

Abbreviations, alphabetically: AE: adverse effects, cis: cisplatin, DLT: dose-limiting toxicity, gem: gemcitabine, Ln.: line of therapy (“2nd” includes 2nd or greater), Mnt.: maintenance therapy after first-line treatment, Met endpt.: whether trial met its prespecified endpoint if applicable, ORR: objective response rate, OS: overall survival, ox: oxaliplatin, PFS: progression-free survival (PFSX indicates X-month PFS rate), Ph: phase. * Measured from first study treatment; mPFS 9.9, mOS 21.4 mo. from first chemotherapy.

**Table 3:** Selected ongoing clinical trials involving immunotherapy in BTCs.

NCT/Name	Ph.	Treatment	Line	Prim. Endpoint	Status	Est. Comp.
Immune-checkpoint-inhibitor-only monotherapy and combination therapy						
NCT05297903	II	XmAb20717 (vudalimab)	2nd	ORR	Recruiting	12/2024
Immune checkpoint inhibitors plus chemotherapy						
NCT03260712 (ABC-09/EORTC-1607)	II	Gem/cis + pembrolizumab	1st	PFS6	Active, not rec.	6/2023
NCT04172402	II	Gem/S-1 + nivolumab	1st	ORR	Active, not rec.	12/2024
NCT04191343	II	Gem/ox + toripalimab	1st	ORR	Recruiting	6/2024
NCT03478488	III	Gem/ox + envafolimab (KN035) Gem/ox	1st	OS	Recruiting	7/2024
NCT03785873	I/II	5-fluorouracil/nal-irinotecan + nivolumab	2nd	DLT (I), PFS (II)	Active, not rec.	5/2023
NCT04308174 (DEBATE)	II	Gem/cis + durvalumab (NA) + durvalumab (A) Gem/cis (NA) + durvalumab (A)	NA + A	R0 resection	Active, not rec.	12/2023
NCT04333927 (ACCORD)	II	Camrelizumab + capecitabine + radiation Observation	A	OS	Active, not rec.	6/2024
NCT05239169	II	Capecitabine + durvalumab + tremelimumab Durvalumab + tremelimumab	A	RFS12	Recruiting	12/2024
Immune checkpoint inhibitors plus molecularly targeted therapy						
NCT04941287 (ETCTN 10476)	II	Atezolizumab + varlilumab + cobimetinib Atezolizumab + varlilumab	2nd	ORR, PFS	Active, not rec.	9/2023
NCT04056910	II	Ivosidenib + nivolumab	2nd	ORR, PFS6	Recruiting	5/2026
NCT05749900 (HERBOT)	I/II	Trastuzumab + nivolumab + gem/cis	1st	Dosing (I), ORR (II)	Not yet rec.	2/2027
NCT04306367	II	Olaparib + pembrolizumab	2nd	ORR	Active, not rec.	12/2023
NCT03991832	II	Olaparib + durvalumab	2nd	ORR, DCR	Recruiting	3/2025
NCT04298008	II	AZD6738 (ceralasertib) + durvalumab	2nd	DCR	Recruiting	12/2024
Immune checkpoint inhibitors plus antiangiogenics						
NCT04506281	II	Gem/ox+ toripalimab + lenvatinib (NA) + capecitabine (A) Capecitabine (A) only	NA + A	EFS	Recruiting	8/2023
NCT05254847	II	Capecitabine + lenvatinib + tislelizumab	A	DFS12	Recruiting	12/2024
NCT05342194	III	Gem/ox or gem/cis + toripalimab + Lenvatinib Gem/ox or gem/cis + toripalimab Gem/ox or gem/cis	1st	OS	Not yet rec.	5/2027
NCT05742750	I/II	Gem/cis + camrelizumab + apatinib	1st	DLT (I), ORR (II)	Not yet rec.	12/2024
NCT05820906	II	Gem/cis + cadonilimab + regorafenib	1st	ORR	Not yet rec.	9/2025
NCT05775159 (GEMINI- Hepatobiliary)	II	MEDI5752 (volrustomig) MEDI5752 + lenvatinib MEDI5752 + bevacizumab	Any	ORR	Recruiting	11/2025
NCT05052099 (COMBATBIL)	I/II	mFOLFOX6 + atezolizumab + bevacizumab	2nd	ORR	Recruiting	6/2024
Other						
NCT04057365	II	DKN-01 + nivolumab	2nd	ORR	Recruiting	8/2024
NCT05849480	I/II	Capecitabine/oxaliplatin + pembrolizumab + CDX-1140	2nd	Dosing (I), ORR and PFS6 (II)	Not yet rec.	-
See Section 3.5 of the text for a list of ongoing CAR-T therapies						

Abbreviations, alphabetically: A: adjuvant, cis: cisplatin, DCR: disease control rate, DFS: disease-free survival (DFSX indicates X-month DFS), DLT: dose-limiting toxicity, EFS: event-free survival, gem: gemcitabine, Est. Comp.: estimated completion date, NA: neoadjuvant, ORR: objective response rate, OS: overall survival, ox: oxaliplatin, PFS: progression-free survival (PFSX indicates X-month PFS rate), Ph.: phase, rec.: recruiting, RFS: recurrence-free survival (RFSX indicates X-month RFS rate).

In contrast, the single-center, open-label phase II NCT03046862 trial studied three different configurations: (1) gemcitabine/cisplatin followed by cisplatin plus durvalumab and tremelimumab; (2) gemcitabine/cisplatin plus durvalumab; and (3) gemcitabine/cisplatin plus durvalumab and tremelimumab, with the primary endpoint of objective response [47,66]. The ORR was 50% in group 1, 72% in group 2, and 70% in group 3. While there was not a statistical comparison between the groups, this suggested the utility of starting immunotherapy up-front and the limited benefit of adding doublet immunotherapy compared to adding monotherapy alone. While the baseline PD-L1 did not have a relationship with the outcome, the changes in PD-L1 in response to treatment did: Tumors that downregulated PD-L1 expression after the first cycle of chemoimmunotherapy had a worse prognosis, and this difference was statistically significant. Furthermore, the subset with increased PD-L1 staining in the immune cells in response to treatment corresponded with better outcomes. These excellent results thus laid the foundation for the phase III TOPAZ-1 trial, which advanced the second treatment regimen from this trial.

TOPAZ-1 formally compared gemcitabine/cisplatin with durvalumab to gemcitabine/cisplatin with placebo [6]. Chemotherapy plus immunotherapy was administered for eight cycles, after which durvalumab (or placebo) was continued as maintenance therapy. TOPAZ-1 met its primary endpoint with a median OS of 12.8 months in the chemoimmunotherapy arm vs. 11.5 months in the standard-of-care arm [6] (subsequently updated to 12.9 and 11.3 months, respectively [67]). Notably, there was no separation of the chemoimmunotherapy and chemotherapy OS and PFS curves until after the 6-month mark, while the subsequent benefit of chemoimmunotherapy persisted thereafter (the OS hazard ratio for the time up to 6 months was 0.91 with a 95% confidence interval of 0.66–1.26 and after 6 months was 0.74 [0.58–0.94]). This suggested a delayed but durable benefit of adding immune therapy in a subset of patients. The ORR was 26.7% for chemoimmunotherapy and 18.7% for chemotherapy alone, similar to the gemcitabine/cisplatin arm in the ABC-02 trial and considerably lower than the preceding phase II trial detailed above. The rates of grade 3 or 4 adverse effects were similar between the treatment groups, affecting 75.7% of the durvalumab group and 77.8% of the placebo group. The subgroup analysis of patients with a PD-L1 tumor area positivity of <1% (PD-L1-negative) or ≥1% (PD-L1-positive) showed similar benefits in both populations, again suggesting that baseline PD-L1 status may not be a useful biomarker for predicting the response to the addition of immunotherapy. The change in PD-L1 expression was not assessed in this trial. The TOPAZ-1 results led to the FDA approval of durvalumab in combination with gemcitabine and cisplatin in the first line for advanced/metastatic BTCs in September 2022 [68]. 

Recently, results from KEYNOTE-966 were announced, with a similar treatment design comparing gemcitabine/cisplatin with or without pembrolizumab [46]. Important contrasts with TOPAZ-1 included the continuation of gemcitabine as part of the maintenance regimen alongside pembrolizumab or placebo, as well as a greater inclusion of sites outside of East Asia. As with TOPAZ-1, the proportions of patients experiencing grade 3/4 adverse effects were similar between the two arms (70% with pembrolizumab and 69% with placebo). KEYNOTE-966 met its primary endpoint with a median OS of 12.7 months with chemoimmunotherapy vs. 10.9 months in the standard-of-care arm; this benefit was similar between the PD-L1-negative and -positive cases, defined as a CPS of <1% or ≥1%, respectively. The ORR for both treatment arms was ~29%. Together, TOPAZ-1 and KEYNOTE-966 solidified the role of immune checkpoint blockade in combination with chemotherapy as a first-line management strategy of advanced BTCs, regardless of PD-L1 status.

Multiple ongoing first-line trials are investigating other ICI monotherapies in combination with chemotherapy. The novel subcutaneously delivered single-domain anti-PD-L1 antibody envafolimab (KN035 [69]) is being studied in a randomized phase III trial (NCT03478488), which is comparing gemcitabine/oxaliplatin with or without envafolimab. Ongoing phase II trials include NCT04191343 (toripalimab plus gemcitabine/oxaliplatin), NCT03796429 (toripalimab plus gemcitabine and S-1), and NCT04172402 (nivolumab plus gemcitabine and S-1).

With the arrival of single-agent immune checkpoint blockade as a component of the first-line treatment of advanced/metastatic BTCs, it will be important to define the role of immunotherapy beyond the first line, noting that most of the second-line ICI-only trials detailed in Section 3.1 were conducted when chemotherapy alone was still considered a standard first-line therapy. A phase I/II trial (NCT03785873) is exploring the combination of standard second-line chemotherapy (5-fluorouracil plus nab-irinotecan) with nivolumab but notably excludes patients who previously received anti-PD-1 or anti-PD-L1 therapy. An important question that remains unanswered is whether there is a role for the continuation of first-line immunotherapy beyond progression on initial chemoimmunotherapy, and whether the escalation of immunotherapy via ICI doublet or an alternate immunotherapy modality (see Section 3.5) might be sequenced after first-line chemoimmunotherapy. The phase II NCT04298008 trial will help to explore this space by investigating treatment with durvalumab in combination with AZD6738 (ceralasertib), an oral ATR inhibitor that modulates the DNA damage response [70], as a second-line treatment for patients with advanced BTCs that have specifically progressed on first-line ICI-containing therapy. This approach to restoring the response to ICIs has previously shown promise in melanoma that has progressed on first-line anti-PD-1 therapy [71].

In summary, there are now two phase III trials confirming the benefit of adding immunotherapy—durvalumab or pembrolizumab—to standard first-line chemotherapy, firmly establishing this approach as the new standard of care. There is currently no guidance on choosing between durvalumab or pembrolizumab in this setting. A subset of patients appears to derive durable benefits from the addition of ICI monotherapy to first-line chemotherapy, but a biomarker for selecting these patients has not yet been identified; neither TOPAZ-1 nor KEYNOTE-966 saw any trend suggesting that the use of PD-L1 is a predictive biomarker. Thus, further follow-up studies are needed to identify alternative biomarkers that may help to predict the response to adding ICIs to chemotherapy for BTCs. Finally, further research into the role of immunotherapy beyond progression on first-line chemoimmunotherapy, which was not a common practice when most of the second-line immunotherapy trials were devised, will be needed.

### 3.3. ICIs in Combination with Molecularly Targeted Therapeutics

#### 3.3.1. Current Landscape of Molecularly Targeted Therapeutics in BTCs

In recent years, there has been a revolution in the use of molecularly targeted agents for BTCs, for which comprehensive molecular testing is now widely recommended for all unresectable or metastatic cases. The use of molecularly targeted therapy has been most apparent in the management of iCCA, in which *FGFR2* alterations (chiefly *FGFR2* fusions and rearrangements) and *IDH1/2* mutations occur in ~11–45% and ~5–36% of cases, respectively [10,72]. Phase II data resulted in the accelerated FDA approval of infigratinib (discontinued by the manufacturer in 2023) [73], pemigatinib [74], and futibatinib [75] in the second-line setting for BTCs bearing an *FGFR2* fusion/rearrangement. Ivosidenib received full FDA approval as a subsequent-line therapy for *IDH1*-mutant biliary tract cancers based on the ClarIDHy phase III trial, which met its primary endpoint with a median PFS of 2.7 mo. compared to 1.4 mo. for the placebo; the median OS was 10.3 mo. vs. 7.5 mo. [76,77]. Other gene alterations are also potentially actionable in BTCs, with *KRAS* mutations appearing more frequently in eCCAs and *HER2* amplification/overexpression occurring more often in GBAs [36]. The targeting of HER2 positivity with trastuzumab plus pertuzumab and *BRAF* V600E mutations with dabrafenib plus trametinib can be considered in subsequent-line therapies, and KRAS-G12C small-molecule inhibitors are being studied in various GI malignancies, including BTCs, though this mutation is relatively rare outside of non-small-cell lung cancer.

While molecularly targeted therapeutics are currently being used predominantly in the second line in patients fit for chemotherapy, and therefore, at present, likely to be sequenced after gemcitabine/cisplatin plus immunotherapy, there are trials underway (e.g., FIGHT-302, comparing pemigatinib vs. gemcitabine/cisplatin) that have the potential to move actionable mutations to the first-line setting [78]. Clinical trials have now begun to address the question of whether there may be a benefit to combining such targeted small-molecule therapeutics with immune checkpoint blockade. The short-lived responses to some targeted monotherapies serve as an impetus to explore the potential benefits of a combination therapy approach, with immunotherapy being particularly attractive for patients who are ineligible for chemotherapy in advanced disease.

#### 3.3.2. IDH1-Targeted Therapies Plus ICI

*IDH1* mutations are seen in iCCA as well as other malignancies, such as acute myeloid leukemia and gliomas. Since *IDH1* mutations can promote an immunosuppressive phenotype, there may be potential for synergy between IDH1 inhibition and ICIs [79]. Given the relatively modest benefit of IDH1-targeted monotherapy seen in the ClarIDHy trial, combination approaches to maximize the benefit of targeting mutant *IDH1* are needed. A phase II trial (NCT04056910) is investigating ivosidenib in combination with nivolumab as a subsequent treatment for advanced solid tumors, including BTCs. Additionally, a phase Ib/II trial (NCT03684811) of FT-2102, also known as olutasidenib, is underway. While the BTC cohort will only be treated with FT-2102 alone or in combination with gemcitabine/cisplatin, there is an HCC cohort that will be treated with the combination of FT-2102 plus nivolumab that can at least shed light on the tolerability of this regimen for possible future investigation in BTCs.

#### 3.3.3. FGFR2-Targeted Therapies Plus ICI

As introduced in Section 2.1, FGFR signaling promotes an immunosuppressive phenotype through downstream effects on both tumor cells themselves and on immune cells including T cells and macrophages. For *FGFR*-altered cancers, Part 3 of the FIGHT-101 phase I/II trial (NCT02393248) included a number of combination therapies with pemigatinib, including dose-finding and dose expansion cohorts of patients treated with pemigatinib plus pembrolizumab or retifanlimab (another anti-PD-1 antibody). The pemigatinib plus pembrolizumab combination was reported to be tolerable with preliminary antitumor activity, though the number of patients who had BTCs out of 23 total patients was not reported [48]. This combination was initially set to be further investigated as a first-line therapy in the phase II FIGHT-205 trial (NCT04003610) in cisplatin-ineligible urothelial carcinoma with *FGFR3* alterations but was terminated for non-medical reasons. A phase II trial of futibatinib plus pembrolizumab (NCT04601857) including cohorts of patients with or without *FGFR* alterations (*FGFR3* mutations or *FGFR1-4* rearrangements) is ongoing. While this is obviously a different disease altogether, insights into the performance and tolerability of this combination could be relevant to *FGFR2*-altered BTCs in the future. 

#### 3.3.4. MAP-Kinase-Pathway-Targeted Therapies Plus ICI

Immunotherapy has been investigated in conjunction with the MEK inhibitor cobimetinib in BTCs. While, to date, MEK inhibitors have conventionally been used in combination with BRAF inhibitors to reinforce the blockade of the MAP kinase (MAPK) growth signaling pathway in BRAF V600E-mutant cancers (including BTCs in the ROAR basket trial [48]), the rationale, in this case, is to use MAPK blockade for immune modulation in addition to the suppression of tumor growth, regardless of *BRAF* or *KRAS* mutational status. Multiple preclinical studies have shown the promotion of T cell function and anti-tumor activity with MAPK inhibition—particularly MEK inhibition—in combination with immunostimulatory therapeutics such as PD-1 or PD-L1 checkpoint inhibition [80,81,82]. While MEK inhibition on its own exerts tumor-intrinsic effects but also a degree of impairment of T cell activation, the latter effect has been shown to be reversed through stimulatory T cell interventions [83]. These findings laid the foundation for a randomized phase II trial examining atezolizumab with or without cobimetinib, which showed a statistically significantly improved median PFS of 3.7 months for the combination vs. 1.9 months with atezolizumab alone [49]. While the disease control rate was 47% in the combination arm, the ORR was only 3%. Deeper investigation showed that while there was evidence of immunomodulation, such as an increase in the CD8^+^ T cell to regulatory T cell ratio and a decrease in circulating growth factors such as PDGF-BB that appeared to be associated with better overall survival [84], there could still be an impairment of T cell priming and activation despite the presence of anti-PD-L1 therapy. The ongoing ETCTN 10476 phase II randomized trial was therefore designed to investigate the combination of atezolizumab plus varlilumab, a CD27 T cell agonist (see also Section 3.5), with or without cobimetinib, in an effort to further tip the balance of the T cell response in BTCs [85].

#### 3.3.5. EGFR Blockade Plus ICI

The epidermal growth factor receptor (EGFR) plays an important role in many cancers through downstream signaling via the MAPK pathway (see Section 3.3.4) as well as the PI3K/Akt/mTOR pathway. While EGFR mutations are uncommon in BTCs, EGFR is robustly expressed in the majority of BTCs while not being present in non-malignant biliary tract conditions [86]. Therefore, EGFR has been explored as a molecular target using both EGFR-targeted small-molecule tyrosine kinase inhibitors (TKIs) and anti-EGFR antibodies. The randomized phase II PICCA trial determined that there was no benefit in ORR, PFS, or OS with the addition of panitumumab to gemcitabine/cisplatin in KRAS wildtype BTCs [87]. Similarly, the randomized phase II BINGO trial did not see an enhancement of chemotherapy (gemcitabine/oxaliplatin) with the addition of cetuximab, though this was a non-comparative trial [88]. For TKIs, a phase III trial comparing gemcitabine/oxaliplatin with or without continuous erlotinib did not see a benefit in PFS with the addition of erlotinib. More recently, a phase Ib trial (NCT00987766) investigated the use of pulsatile erlotinib (5 days every 2 weeks) in combination with gemcitabine/oxaliplatin for advanced BTCs and showed an encouraging 94% DCR at the MTD. 

Preclinical studies in genetically engineered lung tumor models have suggested favorable changes to the immune TME in response to EGFR TKI treatment, such as early increased antigen presentation and CD8+ cytotoxic response, a reduction in Tregs, and the inhibition of M2 macrophages, though this was a dynamic process, with this phenotype disappearing with long-term treatment [89]. This unfavorable end result has been borne out clinically with tumors progressing after EGFR TKIs showing increased PD-L1 and decreased CD8^+^ TILs [90]. Theoretically, the addition of immunotherapy could present an opportunity to reverse this unfavorable long-term phenotype. To our knowledge, there is not currently a BTC trial investigating anti-EGFR therapy with ICIs either concurrently or in sequence; however, EGFR has been tested as a CAR-T target for BTCs, as discussed later in Section 3.5. Furthermore, simultaneous and sequential anti-EGFR and ICI therapy has been investigated in other solid tumor types, particularly non-small-cell lung cancer. With the caveat that these studies have generally been conducted in EGFR-mutant disease (which is more likely to respond to EGFR monotherapy as a comparator), overall, EGFR TKI plus ICI trials in this population have shown disappointing results, with failure to achieve additive effects and concerning increased toxicity signals. These adverse effect profiles included elevated rates of hepatotoxicity with the combination of durvalumab or pembrolizumab with first-generation TKI gefitinib and interstitial lung disease with the combination of durvalumab with third-generation TKI osimertinib, leading to the early closure of both the TATTON and CAURAL trials [90]. While osimertinib, which has also shown toxicity when administered sequentially *after* ICI, is less relevant to BTCs as a mutation-specific EGFR TKI, these trials, together, unfortunately suggest a broader issue with the tolerability of EGFR plus ICI combination therapy. Efforts to explore strategies such as alternate dosing regimens, as mentioned above regarding pulsatile erlotinib dosing, may help to improve tolerability [91].

#### 3.3.6. HER2 Blockade Plus ICI

HER2 amplification/overexpression has been seen in many cancer types; among GI cancers, the rates of such alterations are highest in gastroesophageal cancers, but HER2 alterations are also seen in pancreatic, colorectal, and biliary tract cancers (particularly GBAs). As noted previously, preclinical evidence supports the addition of an ICI to HER2-directed therapy as a way to stimulate HER2-specific immune response and overcome PD-1/PD-L1 signaling as a mode of resistance to trastuzumab. The phase III KEYNOTE-811 trial demonstrated a superior ORR for chemotherapy plus trastuzumab and pembrolizumab compared to chemotherapy plus trastuzumab alone as a first-line therapy for HER2-positive gastroesophageal junction and gastric adenocarcinoma [20]. The phase Ib/II HERBOT trial (NCT05749900) is planned to study gemcitabine/cisplatin plus trastuzumab and nivolumab as a first-line palliative treatment for HER2-positive BTCs, a treatment strategy analogous to KEYNOTE-811.

In addition to the anti-HER2 monoclonal antibodies, small-molecule therapeutics targeting one or more of the HER family proteins have also been developed. For example, the anti-HER2 small-molecule tucatinib is being studied in combination with trastuzumab and chemotherapy or ICI (pembrolizumab) in GI cancers including BTCs (NCT04430738), though BTCs are only being enrolled in the phase 1b non-ICI cohorts [92]. In a case report, a patient with advanced HER2-positive iCCA (PD-L1 negative) was treated with an unconventional sequence of pyrotinib (a multi-targeted small molecule that inhibits EGFR, HER2, and HER4) plus tegafur in the first line; pyrotinib plus lenvatinib and pembrolizumab; and pyrotinib plus bevacizumab and pembrolizumab over a 29-month period, with ongoing stable disease on third-line therapy at the time of publication [93]. While it is difficult to draw conclusions from this complex course, it illustrates the potential of extending anti-HER2 plus ICI treatment with antiangiogenics, which are discussed further in Section 3.4.

#### 3.3.7. PARP Inhibitors Plus ICI

Several trials are investigating the use of PARP inhibitors (PARPi) combined with ICI in advanced BTCs. While PARP inhibitors have traditionally been used in the setting of homologous repair-deficient (HRD) cancers (e.g., BRCA1/2 germline mutations), the rationale for their use in all comers is to promote DNA damage, leading to aberrant protein expression that can serve as tumor neoantigens analogously to TMB-H status. Treatment with a PARP inhibitor also appeared to upregulate PD-L1 expression in animal models of cancer [94]. The phase II BilT-02 trial examined rucaparib plus nivolumab as a maintenance therapy after first-line platinum-based chemotherapy [50]. While this study did not meet its primary endpoint (PFS rate at 4 months), it did show a high DCR of 77% and a preliminary median PFS1 and OS1 (measured from the start of maintenance therapy) of 4.6 and 15.9 months, respectively [51]. Phase II trials underway that are pairing PARPi with ICI include NCT04306367 (olaparib plus pembrolizumab) [95] and NCT03991832 (olaparib plus durvalumab in IDH-mutant disease only). Interim results from the trial investigating olaparib plus pembrolizumab in the second line showed PR in 1/12 patients and stable disease in 4/12 patients [95].

For the HRD subset of BTCs, there are limited reports and no firm evidence to date to support the use of PARPi, though trials are underway and have been reviewed elsewhere [96]. One case report documents a complete response to olaparib plus pembrolizumab after platinum-based induction chemotherapy in a patient with BRCA2-mutant, PD-L1-positive recurrent iCCA [97]. In HRD BTCs, it will be important to better define the role of PARPi and particularly PARPi and immunotherapy: If, for instance, PARPi emerges as a maintenance strategy after a platinum-based regimen as presently used in pancreatic cancer [98], it will also be important to address whether the first-line ICI, such as durvalumab or pembrolizumab, will also continue in the maintenance setting alongside PARPi.

#### 3.3.8. Summary of ICIs in Combination with Molecularly Targeted Therapeutics

In summary, small-molecule inhibitors may be employed in conjunction with immunotherapy in order to augment the response to targeting a particular molecular alteration but also, in an alteration-agnostic manner, in order to prime tumors for response to immunotherapy through immunomodulation and the creation of new tumor neoantigens. These strategies are still actively being investigated in BTCs, as well as other solid tumor types, and while it is apparent that there are complexities beyond the foundational findings established based on preclinical models, the preliminary results in clinical trials have shown some promise.

### 3.4. ICIs in Combination with Anti-Angiogenic Therapeutics

While anti-angiogenic therapeutics are infrequently used in the present management of BTCs, angiogenesis is not only an integral process in BTC development but also established to suppress tumor immunity. VEGF receptors are not limited to endothelial cells alone, in which they promote growth and vascular permeability, but are also expressed on tumor cells themselves and a variety of immune cells, including dendritic cells, macrophages, and T lymphocytes [24]. As noted in Section 2.1, pro-angiogenic factors can contribute to reduced immune response via the engagement of Tregs, MDSCs, and TAMs [99]. This provides the rationale for testing anti-angiogenic drugs, including bevacizumab (anti-VEGF-A), ramucirumab (anti-VEGFR2), and the TKIs lenvatinib and regorafenib (targeting a number of receptors, including VEGFR1/2/3), in combination with ICIs in BTCs. While these targets are related, the therapeutic effects differ to a degree, since the blockade of various VEGF receptors blocks their signaling in response to various VEGF isoforms, and the inhibition of VEGF-A blocks signaling beyond VEGFR receptors, e.g., through neuropilins [100].

Several combinations of antiangiogenic and ICI therapies have been investigated, typically as subsequent therapies. The anti-VEGFR2 antibody ramucirumab, more commonly utilized in the management of other GI malignancies such as gastroesophageal adenocarcinoma and hepatocellular carcinoma, has very limited activity as a monotherapy in the second-line setting for BTCs, with an ORR of only 1.7% and median PFS of 3.2 months [101]. The combination of ramucirumab and pembrolizumab was then studied in the phase Ia/Ib JVDF trial (NCT02443324) [55]. In previously treated advanced BTCs, this regimen was reasonably well-tolerated but did not extend OS compared to historical second-line regimens: the ORR was 4%, the mPFS was 1.6 months, and the mOS was 6.4 months, numerically similar to the performance of FOLFOX in the second line. Using another pairing of anti-VEGFR therapy with an ICI, the multicenter single-arm phase II REGOMUNE trial (NCT03475953) investigated the oral multikinase inhibitor regorafenib plus avelumab in the second line for advanced/metastatic BTCs [54]. The ORR was 13.8% and the DCR was 51.7%, with an mPFS of 2.5 months and mOS of 11.9 months. The single-arm phase II LEAP-005 trial (NCT03797326) studied lenvatinib in combination with pembrolizumab and included a cohort to whom this was applied as a second-line therapy for advanced BTCs [53]. The BTC cohort of LEAP-005 included 31 patients, and the primary endpoint of ORR was 10%, including all PRs, while the DCR was 68%. The phase II GEMINI-Hepatobiliary trial (NCT05775159) is currently studying volrustomig (MEDI5752), a bispecific anti-PD-1/anti-CTLA-4 ICI, alone or in combination with lenvatinib or bevacizumab in HCCs and BTCs.

There have also been multiple studies augmenting the antiangiogenic plus ICI combination with chemotherapy for the first-line setting. Importantly, the addition of an antiangiogenic alone to first-line chemotherapy has not been shown to improve outcomes, with a study investigating ramucirumab in combination with gemcitabine/cisplatin not showing any improvement in PFS compared to chemotherapy alone [102]. However, given the mechanisms of interplay between antiangiogenics and chemotherapy with the immune microenvironment, as described above and in Section 3.2, there could be benefits to a three-pronged approach combining chemotherapy, antiangiogenics, and immunotherapy. The single-site, single-arm phase II NCT03951597 trial studied lenvatinib and toripalimab with concurrent gemcitabine/oxaliplatin for previously untreated unresectable iCCA, with 40% of cases having distant metastatic disease [57]. This aggressive regimen resulted in a very promising ORR of 80% and DCR of 93.3%; the mPFS was 10.0 months and mOS was 22.5 months. Grade 3 and 4 AEs were reported in 57% and 10% of patients, respectively, a profile comparable to the those of the major chemotherapy plus ICI trials. The present follow-up phase III trial (NCT05342194) in iCCA will randomize patients to three arms: toripalimab plus lenvatinib plus chemotherapy, (B) toripalimab plus chemotherapy, and (C) chemotherapy alone, where the chemotherapy may be either gemcitabine/oxaliplatin or gemcitabine/cisplatin. A similar quadruple-therapy approach is also being studied in a phase Ib/II study (NCT05742750), which adds the selective VEGFR2 TKI apatinib and camrelizumab to the gemcitabine/cisplatin backbone. Unlike the toripalimab/lenvatinib plus chemotherapy trials, this study includes all BTC subtypes. An upcoming single-center, single-arm phase II trial (NCT05820906) is combining regorafenib and gemcitabine/cisplatin with the bispecific ICI antibody cadonilimab (anti-PD-1 and anti-CTLA-4), escalating the immunotherapy component of this multimodality approach. Finally, the IMBrave-151 phase II trial randomized patients to gemcitabine/cisplatin/atezolizumab plus bevacizumab or gemcitabine/cisplatin/atezolizumab plus placebo [56]. The median PFS was 8.4 months with bevacizumab and 7.9 months without, with 6-month PFS rates of 78% and 63%, respectively. OS data are not yet available at the time of writing, and a statistical comparison between the treatment groups was not part of the trial’s design. Importantly, the addition of bevacizumab did not appear to contribute to additional toxicity. The ongoing COMBATBIL (NCT05052099) trial is studying second-line doublet chemotherapy (FOLFOX) with the same combination of atezolizumab and bevacizumab.

Taken together, these trials suggest the limited activity of antiangiogenic plus ICI combined therapy without chemotherapy as a second-line treatment. Meanwhile, encouraging phase II data on the efficacy and tolerability of first-line chemoimmunotherapy plus anti-angiogenic therapy have prompted further investigation of this aggressive approach in multiple phase II and phase III trials.

### 3.5. Other Immunotherapies

One of the most extensively studied immunotherapies outside of conventional immune checkpoint monoclonal antibodies in BTCs is bintrafusp alfa (M7824). Bintrafusp alfa is a bifunctional fusion protein targeting TGF-beta and PD-L1, where the TGF-βRII domain is used as a “trap” to sequester TGF-beta, while the anti-PD-L1 monoclonal antibody domain functions as an ICI. Bintrafusp alfa was initially studied as a monotherapy in the second line for advanced BTCs, with promising results showing an objective response rate of 20% and a median PFS and OS of 2.5 months and 12.7 months, respectively [59]. Unfortunately, the follow-up INTR@PID BTC 047 phase II trial (NCT03833661) in the second line for advanced/metastatic BTC showed an ORR of just 10.7%, failing to meet its prespecified primary endpoint threshold of having a lower bound of the 95% confidence interval exceeding 10% [60]. The median PFS was 1.8 months, while the median OS was 7.6 months. The focus thus switched to investigating bintrafusp alfa in the context of combination therapy. The INTR@PID BTC 055 phase II/III trial (NCT04066491) intended to (1) investigate the combination of gemcitabine/cisplatin with bintrafusp alfa in the first-line setting followed by bintrafusp alfa maintenance, and (2), in the randomized portion, to compare gemcitabine/cisplatin with bintrafusp alfa or placebo, with the primary endpoint of OS [103]. This trial was discontinued in 2021 based on the low likelihood of achieving its primary endpoint [104]. A phase I trial (NCT04708067) is ongoing that is instead exploring the safety of bintrafusp alfa in combination with hypofractionated radiation therapy in iCCA. The overall disappointing results from bintrafusp alfa may, in part, be due to the complexities of TGF-beta signaling and possible overestimation of its role in immune evasion [105].

Agonist antibodies aiming to enhance immune responses through targets outside of classical ICI are also being explored. In contrast to classic ICI, in which antibodies targeting PD-1, PD-L1, or CTLA-4 block inhibitory signaling via these checkpoint molecules, such agonists are designed to mimic the natural ligands of their targets and thus potentiate stimulatory signaling. The agonism of the co-stimulatory receptor CD27 on T cells induced by varlilumab is being explored in combination with MEK inhibition and ICI, as described in Section 3.3.4 above. The agonism of CD40 using antibodies that act like CD40L, normally found on CD4^+^ T helper cells, can promote dendritic cell activation and help to enhance a cytotoxic T cell response [106]. CDX-1140 is one such CD40 agonist that is currently being studied in combination with capecitabine/oxaliplatin chemotherapy and pembrolizumab in the second line in a phase I/II trial (NCT05849480).

Dickkopf-1 (DKK1) has emerged as an intriguing target due to its role in immunosuppression via Wnt signaling. An anti-DKK1 monoclonal antibody, DKN-01, has been studied in different treatment configurations in advanced BTCs. A phase I trial (NCT02375880) evaluating DKN-01 in the first line alongside gemcitabine/cisplatin showed an ORR of 21.3% with an mPFS of 8.7 months, concluding that there did not appear to be an additional benefit over the historical performance of gemcitabine/cisplatin [61]. Based on the rationale of augmenting traditional ICI with DKK1 blockade to improve immune response, an ongoing phase II trial (NCT04057365) is investigating DKN-01 plus nivolumab in the second line for advanced/metastatic BTCs.

Cancer vaccines have been explored in BTCs using WT1 and MUC1 as antigens due to their ubiquity: WT1 is mutated in ~80% of BTCs, while MUC1 is overexpressed in ~90% [107]. A phase I trial of a WT1 peptide vaccine plus gemcitabine in gemcitabine-naïve advanced BTCs with HLA-A 0201, HLA-A 0206, and/or HLA-A 2402 showed a 2-month disease control rate of 50% and median OS of approximately 10 months, and 59% of patients were found to have WT1-specific T cells [64]. A separate phase I trial of an MUC1 peptide vaccination demonstrated safety, but only one of eight total patients had stable disease, with all other patients having progressive disease [65]. MUC1 has also emerged as a potential target for chimeric antigen receptor T cell therapy, as described below. In the future, personalized cancer vaccines for BTCs may be possible, as suggested by a recent phase I trial studying individualized neoantigen mRNA vaccines in pancreatic cancer in combination with atezolizumab and chemotherapy [108].

Finally, more recently, adoptive cell therapies have also been investigated in BTCs. In particular, chimeric antigen receptor T cell (CAR-T) therapies are of interest, with preclinical data to support varied extracellular targets with preferential expression on tumor cells, including CD133 (estimated to be expressed in ~68% of BTCs) [109], EGFR (50–100%) [62], MUC1 (50–87%) [110], and integrin α_v_β_6_ (73%) [111]. There is limited trial experience for CAR-T treatment in BTCs. A phase I study (NCT01869166) of cyclophosphamide and nab-paclitaxel conditioning followed by the administration of autologous EGFR-targeted second-generation CAR-T cells in EGFR-positive advanced BTCs resulted in 1/17 patients with CR and 10/17 patients with stable disease [62]. The adverse effects were typical for CAR-T treatment. A number of additional CAR-T trials for BTCs are currently open, targeting mesothelin (NCT05568680, NCT05373147, and NCT05779917), HER2 (NCT04650451 and NCT04660929), MUC1 (NCT03633773), GPC3 (NCT04951141), and CEA (NCT05415475). Beyond CAR-T therapy, a phase I/IIa trial (NCT03358849) studying allogeneic NK cells (SMT-NK) plus pembrolizumab in the second line for advanced/metastatic BTCs demonstrated an ORR of 17.4% in the full analysis and 50.0% in the per-protocol analysis [63]. A direct comparison of this combination approach to pembrolizumab monotherapy is planned.

In summary, a number of immunotherapies outside of conventional ICI have been or are being explored in BTCs, including dual ICI and TGF-beta trapping using bintrafusp alfa, immunostimulatory agonists targeting CD27 or CD40, anti-DKK1 therapeutics that target Wnt signaling, cancer vaccines, and adoptive cell therapies including CAR-T and allogeneic NK cells.

## 4. Immunotherapy in the Adjuvant or Neoadjuvant Setting

While the surgical resection of BTCs, when feasible, represents the primary curative modality for managing these diseases, the rates of post-operative recurrence are unacceptably high at 50–70% [112]. The current standard of care for resectable BTCs is adjuvant capecitabine, based on the phase III BILCAP study that compared capecitabine to surveillance. In the long-term analysis, the intention-to-treat mOS was 49.6 mo. for capecitabine vs. 36.1 mo. in the surveillance group (HR 0.84), with this difference notably not reaching statistical significance. The per-protocol analysis was statistically significant, with an OS hazard ratio of 0.74 [113]. Critically, 5-year recurrence-free survival (RFS) was just 34% in the capecitabine arm and 31% in the observation arm. Two phase III studies examining gemcitabine-based adjuvant chemotherapy regimens, BCAT (limited to eCCA) [114] and PRODIGE 12-ACCORD 18 [115], were both negative. 

To date, there has not been a phase III trial to support the use of neoadjuvant or perioperative treatment for resectable BTCs. Neoadjuvant chemotherapy is generally only employed in select cases, for instance, as part of a liver transplantation protocol for perihilar cholangiocarcinoma [116]. The role of perioperative chemotherapy for GBA is being studied in the phase II/III OPT-IN trial (ECOG-ACRIN EA2197, NCT04559139), where patients will either receive chemotherapy solely in the adjuvant setting or the same duration of chemotherapy split between the neoadjuvant and adjuvant periods [117].

Perioperative immunotherapy offers the opportunity to harness the adaptive immune system in order to reduce recurrence, with preclinical data supporting neoadjuvant therapy as an ideal setting given the larger malignant burden that can serve as a source of tumor antigens in conjunction with immunotherapy [118]. Immunotherapy is playing an increasing role in the perioperative management of other solid malignancies, such as triple-negative breast cancer, non-small-cell lung cancer, melanoma, and urothelial carcinoma [119]. In particular, for hepatocellular carcinoma, the interim analysis of the phase III IMBrave-050 trial recently showed a recurrence-free survival benefit from adjuvant atezolizumab plus bevacizumab, though it remains to be seen if this will improve long-term outcomes [120]. 

Several phase II trials are ongoing, studying the possible incorporation of immunotherapy into the adjuvant treatment of BTCs. All of these build upon the capecitabine backbone established by the BILCAP trial and include NCT05254847 (single-arm capecitabine plus lenvatinib and tislelizumab), NCT04333927 (randomization to camrelizumab plus chemoradiation with capecitabine vs. observation), and NCT05239169 (randomization to capecitabine plus durvalumab and tremelimumab vs. durvalumab and tremelimumab). These trials will help to inform whether there may be a role for the incorporation of ICIs into the adjuvant setting, where the current options are limited and innovative interventions that can boost long-term recurrence-free survival are greatly needed.

Immunotherapy, as a component of neoadjuvant therapy for BTCs, is also being explored in combination with chemotherapy or chemotherapy plus anti-angiogenic therapy. The phase II DEBATE trial (NCT04308174) is randomizing patients to pre-operative gemcitabine/cisplatin with or without durvalumab for up to four cycles, with all groups also receiving six cycles of adjuvant durvalumab [121]. The R0 resection rate is the primary endpoint of this study, while OS, EFS (event-free survival), ORR, and adverse events are the secondary endpoints. The phase II NCT04506281 trial adapts the aggressive regimen of toripalimab plus gemcitabine/oxaliplatin plus lenvatinib previously studied in the advanced/metastatic setting (see NCT03951597 in Section 3.4) as a neoadjuvant regimen for resectable iCCA at high risk of recurrence, compared to no neoadjuvant treatment. All patients will receive adjuvant capecitabine as per the BILCAP regimen. The primary outcome is EFS, while the secondary outcomes are OS, ORR, pCR, and adverse events. These contrasting approaches to perioperative therapy, both considerably intensified compared to the standard-of-care adjuvant capecitabine approach, will help to inform whether there is a role for immunotherapy in the neoadjuvant setting.

For the subset of CCA patients eligible for liver transplant, there is no established role for immunotherapy peri-transplant, and this needs to be approached with caution given the potential for increasing the risk of graft rejection with ICI administration. The risk of allograft rejection has been described in the hepatocellular carcinoma (HCC) literature in both the neoadjuvant and post-transplant settings (e.g., as a management strategy for recurrent disease) [122,123], and ongoing HCC-centric clinical trials investigating safer approaches to using immunotherapy prior to or after liver transplant may help to inform whether there are strategies that could be employed in transplant for CCA in the future.

## 5. Conclusions

Immunotherapy has a proven role in the management of BTCs, with ICIs now considered a standard first-line therapy for advanced or metastatic disease in combination with doublet chemotherapy. Supported by preclinical and clinical observations regarding the role of chemotherapy, targeted therapies, and anti-angiogenic therapies in modulating the immune microenvironment, a multitude of regimens combining ICIs with one or more of these agents are currently being investigated, with mixed results thus far. Given the expansion of targeted therapies against a wide array of actionable molecular alterations seen in BTCs, it will be critical to determine which approaches might benefit from concomitant immunotherapy. Newer approaches that harness the immune system outside of conventional ICIs are also being explored and present an opportunity for farther-reaching or more nuanced control of the immune response, though many of these therapeutics remain in the early stages of development. Adjuvant therapy and neoadjuvant therapy remain controversial areas of BTC management, and immunotherapy is also being studied in this context, where effective methods for reducing disease recurrence are greatly needed. Thus, important inroads have been made in the incorporation of immunotherapy into BTC management, and ongoing trials may expand its use in the near future.
